# CRISPR/Cas9-targeted mutagenesis of *SlCMT4* causes changes in plant architecture and reproductive organs in tomato

**DOI:** 10.1093/hr/uhac081

**Published:** 2022-04-11

**Authors:** Xuhu Guo, Jianguo Zhao, Zhiwen Chen, Jun Qiao, Yongfang Zhang, Hong Shen, Zongli Hu

**Affiliations:** School of Life Sciences, Shanxi Datong University, Datong 037009, China; Protected Agricultural Technology Research and Development Center, Shanxi Datong University, Datong 037009, China; Institute of Carbon Materials Science, Shanxi Datong University, Datong 037009, China; Institute of Carbon Materials Science, Shanxi Datong University, Datong 037009, China; Institute of Carbon Materials Science, Shanxi Datong University, Datong 037009, China; School of Life Sciences, Shanxi Datong University, Datong 037009, China; School of Life Sciences, Shanxi Datong University, Datong 037009, China; Laboratory of Molecular Biology of Tomato, Bioengineering College, Chongqing University, Chongqing 400044, China

## Abstract

DNA methylation participates widely in the regulation of gene expression in plants. To date, the regulation and function of DNA methylation is still unknown in tomato plants. Here, we generated *SlCMT4* mutants using the CRISPR-Cas9 gene editing system. We observed severe developmental defects in CRISPR-Cas9-mediated *SlCMT4* mutants, including small and thick leaves, increased lateral buds, defective stamens and pistils, small fruit size with reduced setting rate, and defective seed development. The alterations at hormonal levels (IAA, tZR, strigol) were consistent with the multibranching phenotype in *SlCMT4* mutant plants. CRISPR-Cas9-mediated knockout of *SlCMT4* induced the expression of two pollen-specific genes (*PMEI* and *PRALF*) that suppressed the development of pollen wall and pollen tube elongation, which is responsible for irregular and defective pollen. The small-sized fruit phenotype is probably associated with upregulated expression of the *IMA* gene and reduced seeds in the mutant lines. Furthermore, we performed whole-genome bisulfite sequencing (WGBS) of fruits and found that *SlCMT4* knockout reduced genome-wide cytosine methylation. A reduction of methylation was also observed in a 2-kp region of the *IMA* and *LOXB* promoters in the *SlCMT4*-mutant fruits, indicating that the hypermethylation status of the CHH context is critical for the inhibition of *IMA* and *LOXB* promoter activity. Our results show that *SlCMT4* is required for normal development of tomato vegetative and reproductive organs. This study illuminates the function of *SlCMT4* and sheds light on the molecular regulatory mechanism of tomato plant architecture and fruit development and ripening.

## Introduction

Epigenetic regulation is an important part of gene mRNA accumulation and has become a hot spot in current research. DNA methylation plays a critical role in gene imprinting, genome stability, development, and response to the environment [1–3]. DNA methylation is mainly catalyzed by DNA methyltransferases (MTases), including methyltransferase 1 (MET1), chromomethylases (CMTs), domains rearranged methyltransferases (DRMs), and DNA methyltransferase 2 (DNMT2) in plants [4]. *MET1* encodes the DNA methyltransferase that is associated with maintaining CG methylation. Pleiotropic developmental abnormalities are observed in antisense-*MET1* lines of *Arabidopsis* with decreased methylation level, such as smaller plant size, altered leaf size and shape, reduced apical dominance, altered flowering time, and decreased fertility [5, 6]. *Arabidopsis* plants with reduced endogenous expression of the *MET1* gene display severe phenotypes [7–9], whereas homozygously targeted rice plants shows no apparent phenotypic alterations [10]. *Arabidopsis thaliana* embryos exhibit reduced viability and altered planes and numbers of cell division due to dysfunction in *MET1* and *CMT3* [8]. Hypomethylation of DNA in CG sequences is detected in tobacco transgenic lines by expressing an antisense *NtMET1* construct [11]. *NtMETl* transgenic plants with significantly decreased methylation levels of genomic DNA show severe phenotypic defects, including short internodes, small leaves, and abnormal flower morphology [11]. Distinct developmental defects are observed in *slmet1* mutants generated using CRISPR-Cas9 gene editing, including defective inflorescence, small and curly leaves, and parthenocarpy [12].

CHG and CHH (H = A/C/T) methylations are mediated by CMTs and DRMs [2]. A study by Moritoh *et al*. [13] revealed that targeted disruption of rice *OsDRM2* by homologous recombination-mediated gene targeting led to a series of developmental phenotypes in different growth and development stages, such as defective growth, semi-dwarf stature, reduced tiller number, delayed heading or no heading, abnormal panicle and spikelet morphology, and complete sterility [13], whereas no developmental defects were observed in the *drm2* mutants of *Arabidopsis*. *dmt103*, loss-of-function lines in maize (*Zea mays*), shows severe developmental defects in seed morphologies at the reproductive stage, but no morphological alterations at the vegetative developmental stage [14]. In addition, plants with T-DNA insertion of *OsTSD2*, a putative rice methyltransferase gene, display dwarfness and serial alterations in the root zones [15]. Bartee *et al*. [16] reported that *Arabidopsis cmt3* mutants in the pai1C251Y background displayed developmental alterations. These alterations include strongly reduced fluorescence at the seedling stage and partially reduced fluorescence at the plant adult stage, with increased size, decreased bushiness, and increased fertility. Cao and Jacobsen [17] revealed that *cmt3* single mutants and *drm1*, *drm2* double mutants all showed no morphological defects, but *drm1*, *drm2*, *cmt3* triple mutant lines showed pleiotropic alterations in plant development. Recently, Chen *et al*. [18] showed that suppressed expression of the *CMT3* gene through virus-induced silencing (VIGS) in the tomato mutant induced expression of the *CNR* gene and triggered fruit ripening in the epimutant. Some similar effects on the ripening process in *Cnr* tomato mutants are also found through VIGS of the *SlMET1*, *SlDRM7*, and *SlCMT2* genes. In addition, Fieldes *et al*. [19] showed that the level of methylation decreased and flowering time was earlier after treating flax with 5-azaC (5-azacytidine, a methylation inhibitor).

**Figure 1:**
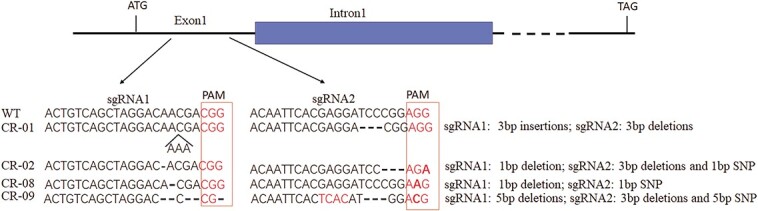
*SlCMT4* gene knockout mediated by the CRISPR-Cas9 system. Schematic map of the sgRNA targeted sites on the genomic regions of *SlCMT4*, and sequencing results of the *SlCMT4* homozygous mutant lines from the T1 generation. The edited exon is shown as lines; the adjacent intron is shown as a blue box. Other exons and introns are shown as dashed lines. PAMs (protospacer adjacent motifs; NGG) are shown in red boxes, black arrows represent insertions, and dashes indicate deletions.

In recent years, the importance of DNA methyltransferases in plant development, transcriptional regulation, and control of metabolic pathways has been increasingly recognized. At present, there are many research reports on genes related to fruit ripening, but there are few research reports that clarify the regulatory mechanism of fruit ripening from the aspect of epigenetics, especially DNA methylation. The epigenetic regulation mechanism of tomato fruit development and ripening remains unclear. Preliminary studies showed that expression of the tomato DNA methyltransferase gene *SlCMT4* was high in floral organs and immature green fruit, but declined during leaf development and fruit ripening [20]. To investigate DNA methylation in tomato (*Solanum lycopersicum* ‘Ailsa Craig’ AC^++^), we applied CRISPR-Cas9-mediated gene knockout to generate targeted disruptants of *SlCMT4*, which encode DNA methyltransferases responsible for *de novo* and non-CG methylation as annotated in *Arabidopsis*. Furthermore, we analyzed the *SlCMT4* mutant lines at morphological, physiological, molecular, transcriptome, and methylome levels. This study enhances our understanding about the role of *SlCMT4* in diverse developmental processes and thus has important theoretical significance.

## Results

### Efficient mutation of the *SlCMT4* gene via the CRISPR-cas9 system

*SlCMT4* (Solyc08g005400.2) was mutated in cv. ‘Ailsa Craig’ using the CRISPR/Cas9 system to confirm its function. The two targeted sites were located on the first exon of the *SlCMT4* gene (Fig. 1). In total, six kanamycin-resistant lines were obtained from the T0 transgenic lines. Among them, four lines (CR-01, CR-02, CR-08, and CR-09) were found to have mutations at the first target sites (CR-01, 3-bp insertions; CR-02, 1-bp deletion; CR-08, 1-bp deletion; CR-09, 5-bp deletion) and the second target sites (CR-01, 3-bp deletions; CR-02, 3-bp deletions and 1-bp SNP; CR-08, 1-bp SNP; CR-09, 3-bp deletion and 5-bp SNP) (Fig. 1). We also obtained four types of *SlCMT4* homozygous mutants from the self-pollinated T0 lines. Through CRISPR-cas9 gene editing and sequencing technology, we obtained four mutant lines, CR-01, CR-02, CR-08, and CR-09 (Fig. 1), among which CR-08 and CR-09 had more obvious phenotypic changes; thus we focused on these two lines as the main studied objects.

### CRISPR-Cas9-mediated knockout of *SlCMT4* promotes branching

**Figure 2:**
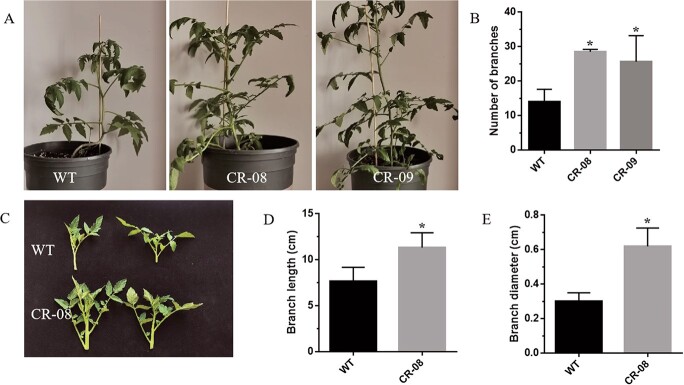
CRISPR/Cas9-targeted mutagenesis of the *SlCMT4* gene induced lateral branch growth in tomato. **a** Seedling (45 days old) morphology of WT, CR-08, and CR-09 lines, from left to right. **b** Lateral branch number in WT and mutant lines (3 months old). **c** Five-day-old lateral buds from WT and mutant lines. **d**, **e** Length (**d**) and diameter (**e**) of lateral buds. Data are means of three biological replicates ± standard deviation. Asterisks indicate statistically significant differences relative to WT, determined using *t*-tests. ^*^P < .05.

**Figure 3:**
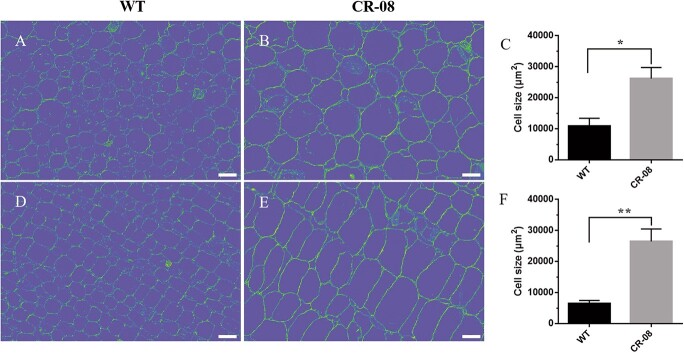
Anatomical analysis of lateral buds from WT (**a**, **d**) and CR-08 (**b**, **e**) lines. **a** and **b** Parenchyma cells in pith from transverse section of lateral buds. **c** Parenchyma cell area was estimated in transverse sections of lateral buds. **d** and **e** Parenchyma cells in pith from longitudinal sections of lateral buds. **f** Parenchyma cell area was estimated in longitudinal sections of lateral buds. Scale bars = 100 μm. The samples were prepared from 5-day-old lateral buds.

**Figure 4:**
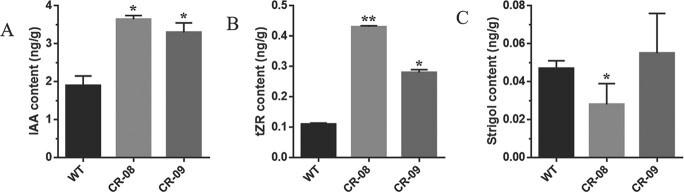
Effect of *SlCMT4* knockout on lateral buds at hormone levels. **a**–**c** Contents of IAA, tZR and strigol, respectively, in lateral buds of WT and mutant lines. Each value represents the mean ± standard deviation of three replicates. Asterisks indicate statistically significant differences relative to WT, determined using *t*-tests. ^*^P < .05, ^**^P < .01.

*SlCMT4* mutant lines CR-08 and CR-09 exhibited increased lateral buds at the seedling stage (Fig. 2a). Statistical analysis showed that the number of lateral buds in mutant CR-08 and CR-09 lines were significantly higher than in wild type (WT) at the mature stage (3 months old) (Fig. 2b). This change in the number of lateral buds was also observed in *SlCMT4* RNAi lines (Supplementary Fig. S1). By comparing lateral buds grown for 5 days (Fig. 2c), we found that the length and diameter of the lateral buds in the *SlCMT4* mutant line CR-08 were obviously larger than those of the WT (Fig. 2d and e). Anatomical data revealed that the parenchyma cells in pith of the lateral buds from the *SlCMT4* gene mutant line became larger in transverse and longitudinal sections (Fig. 3), which was similar to the size alteration of cortical cells in the mutant lines (Supplementary Fig. S2). Given the increased lateral branches phenotype of *SlCMT4* mutant plants, we further studied the effects of *SlCMT4* knockout on lateral branches at hormone levels. The results revealed the content of Indole-3-aceticacid (IAA) and trans-Zeatin-riboside (tZR) in the lateral buds of the *SlCMT4* mutant lines increased (Fig. 4a and b); tZR content was increased ~4-fold in lateral branches of the mutant line CR-08. Consistent with the reduced content of strigol in *SlCMT4* RNAi lines (Supplementary Fig. S3), the content of strigol decreased in the CR-08 line (Fig. 4c).

### Knockout of *SlCMT4* changes leaf morphogenesis

**Figure 5:**
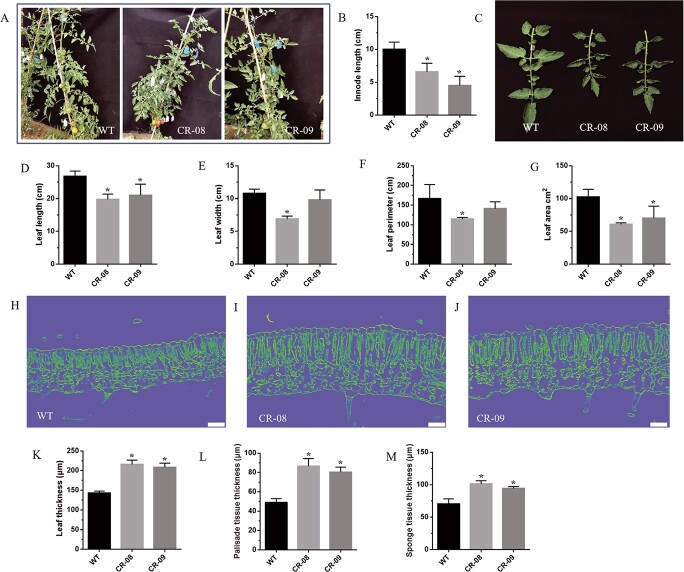
Leaf morphologies of WT and mutant lines. **a** Gross morphologies of WT, CR-08, and CR-09 lines, from left to right. **b** Internode length of 3-month-old WT and mutant lines. **c** Leaf morphologies of WT and mutant lines. **d**–**g** Length, width, perimeter, and area of leaves, respectively, from WT and mutant lines. **h**–**j** Longitudinal sections of leaves from WT and mutant lines (CR-08, CR-09). Scale bars = 50 μm. **k**–**m** Thickness of leaves, palisade tissue, and sponge tissue, respectively, of WT and mutant lines. Each value is the mean ± standard deviation of three replicates. The asterisk indicates a significant difference (*P* < .05) between WT and mutant lines.

The *SlCMT4* mutant lines displayed compact architecture (Fig. 5a) and small internodes (Fig. 5b). The mutant lines also exhibited small leaves (Fig. 5c), which was manifested in the significantly smaller leaf length, width, perimeter, and area (Fig. 5d–g). RNAi suppression of *SlCMT4* also produced a similar leaf phenotype (Supplementary Fig. S4). Longitudinal section analysis of WT and mutant line leaves (Fig. 5h) further revealed that the thickness, palisade, and sponge tissue thickness of the mutant lines increased significantly, and the gap between the palisade and sponge tissue was larger (Fig. 5k–m). These observations illustrated that leaf structure differed at the microscopic level between WT and mutant lines.

### CRISPR-Cas9-mediated knockout of *SlCMT4* affects floral organ morphology and pollen development

**Figure 6:**
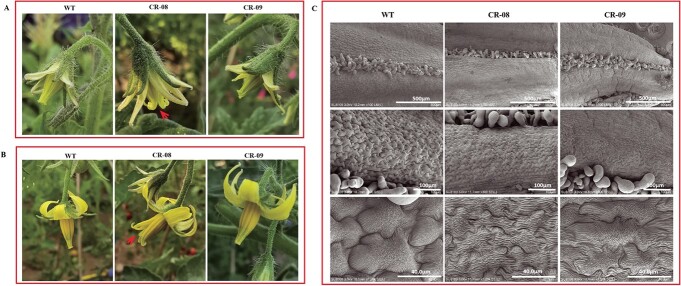
Phenotypes of stamen in *SlCMT4* mutant lines. **a** Flowers with half-open petals. **b** Flowers with fully open petals. Red arrows indicate stamens of CR-08 lines. **c** Scanning electron micrographs of stamens from WT and mutant lines.

**Figure 7:**
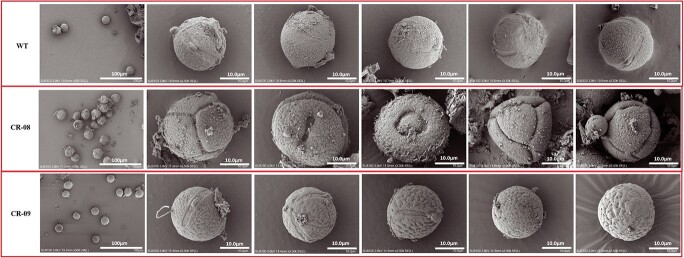
Scanning electron micrographs of pollen from WT and mutant lines (CR-08 and CR-09).

**Figure 8:**
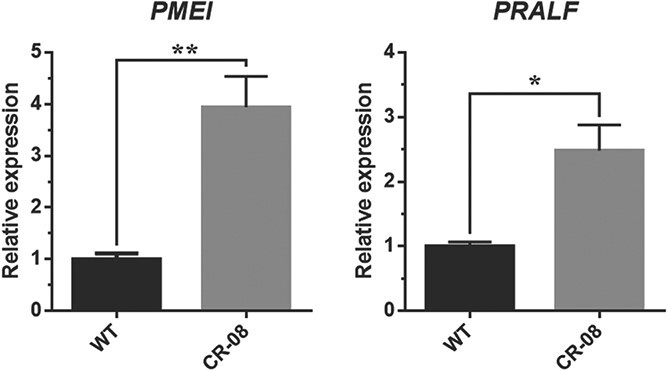
Transcriptional analysis of pollen-specific genes in WT and CR-08 lines. Each value represents the mean ± standard deviation of three replicates. Asterisks indicate a significant difference (^*^P < .05, ^**^P < .01) between WT and CR-08 lines.

The floral organs of CR-08 lines were obviously defective and everted at anthesis (Fig. 6a). Scanning electron microscopy revealed that the stamen cells (Fig. 6c) and pollen grains (Fig. 7) were irregular and defective in the mutant lines. We also studied the expression of tomato pollen-specific genes in CR-08 by quantitative real-time PCR (qRT–PCR) analysis, and found that *PMEI* and *PRALF* were markedly upregulated in the pollen of the mutant line CR-08 (Fig. 8). In addition, the mutant line CR-08 showed short and thick pistils compared with WT (Supplementary Fig. S5).

### CRISPR-Cas9-mediated knockout of *SlCMT4* leads to reduced fruit size

**Figure 9:**
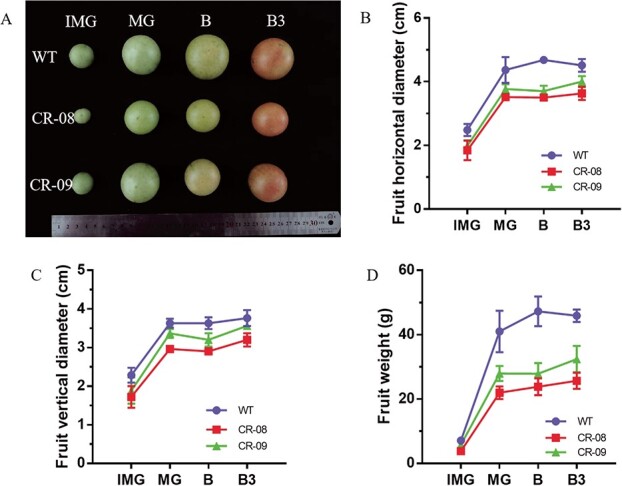
Phenotypes of fruits in the *SlCMT4* mutant lines. **a** Digital photograph of fruits at different developmental stages from WT and mutant lines. **b**–**d** Horizontal diameter, vertical diameter, and weight of fruits, respectively, from WT and mutant lines at different stages. IMG, MG, B, and B3 represent immature green fruit, mature green fruit, fruits at breaker stage, and 3 days after breaker fruit, respectively.

**Figure 10:**
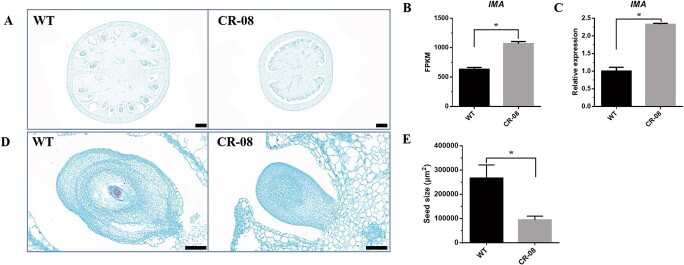
**a** Transverse section of fruit at IMG stage from the WT and CR-08 lines. Bars = 1000 μm. **b** FPKM (fragments per kilobase of transcript per million mapped reads) values of the fruit size-related gene (*IMA*) based on RNA-seq data. **c** Validation of *IMA* expression by qRT–PCR. **d** Seed morphology of IMG fruit. Scale bars = 100 μm. **e** Seed size of IMG fruit. Each value represents the mean ± standard deviation of three replicates. The asterisk indicates a statistically significant difference relative to WT, determined using the *t*-test. ^*^P < .05.

The *SlCMT4* mutant lines displayed small fruit compared with WT (Fig. 9a). The data showed that the horizontal diameter, vertical diameter, and weight of the fruit at different maturity periods [immature green fruit (IMG), mature green fruit (MG), breaker (B), 3 days after breaker (B3)] was decreased in the *SlCMT4* mutant lines (Fig. 9b–d). A similar result was observed in the *SlCMT4*-RNAi plants (Supplementary Fig. S6). Transverse anatomical analysis of fruit at IMG stage showed reduced seeds in the CR-08 lines compared with WT (Fig. 10a). The expression level of the fruit size-related gene *IMA* was upregulated in fruits at IMG stage of the CR-08 line based on RNA-seq (Fig. 10b). qRT–PCR analysis validated that the fruit size-related gene *IMA* was upregulated by ~2.3-fold in fruit at the IMG developmental stage of the mutant lines (Fig. 10c). Besides, seeds from IMG fruit in the CR-08 line were smaller compared with WT (Fig. 10d and e).

### Differentially expressed genes and enrichment analysis

We collected pericarp tissue samples at breaker stage from WT and CR-08 lines to perform a comparative transcriptome analysis. The RNA-seq data were reliable on the basis of Pearson correlation coefficients (>.90) of the three biological replicates (Supplementary Fig. S7A). Two different levels of gene expression were recorded in WT and CR-08 from principal component analysis of six pericarp samples (Supplementary Fig. S7B). We specifically paid close attention to genes that were differentially expressed (DEGs) in the pericarp samples in the CR-08 line. Differential expression of genes was defined using a 1.5-fold change and a *P*-value of <.05 as the cutoffs (Supplementary Fig. S7C). We identified 4102 DEGs, among which 2524 were upregulated and 1578 were downregulated (Supplementary Fig. S7D). Supplementary Fig. S8 lists the results of Gene Ontology (GO) category enrichment analysis using GOseq [21] for DEGs. GO terms associated with critical cellular components were enriched in pericarp of the CR-08 line, including organelle part, membrane-enclosed lumen, macromolecular complex, cell junction, extracellular region, and symplast. Molecular functions that were enriched included structural molecular activity, nucleic acid-binding transcription factor activity, transporter activity, molecular transducer activity, and nutrient reservoir activity. Supplementary Fig. S9 shows that most DEGs were categorized as belonging to functional pathways responsible for the following: (1) metabolism, including carbon metabolism, biosynthesis of amino acids, phenylpropanoid biosynthesis, amino sugar and nucleotide sugar metabolism, and starch and sucrose metabolism; (2) genetic information processing, such as protein processing in the endoplasmic reticulum; and (3) environmental information processing, such as plant hormone signal transduction.

### Transcriptional analysis of fruit ripening-related genes in *SlCMT4* mutants

**Figure 11:**
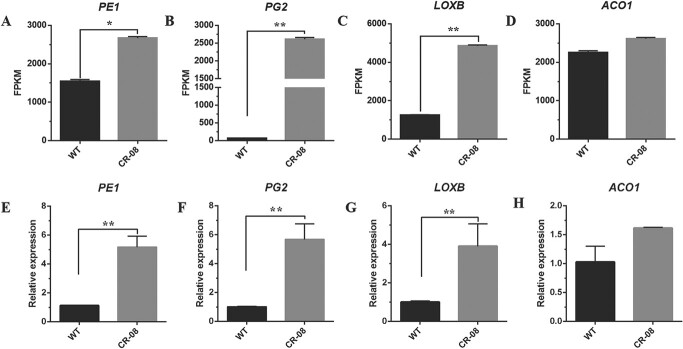
Mutant *SlCMT4* lines had increased transcript levels of ripening-related genes. **a**–**d** FPKM (fragments per kilobase of transcript per million mapped reads) values of fruit ripening-related genes based on RNA-seq data in WT and mutant lines (B stage). **e**–**h** Relative transcription levels of fruit ripening-related genes by qRT–PCR in WT and mutant lines. Each value represents the mean ± standard deviation of three replicates. Asterisks indicate statistically significant differences relative to WT, determined using *t*-tests. ^*^P < .05, ^**^P < .01.

Using WT and CR-08 line fruits (B stage) as materials, transcriptome sequencing technology was used to study the transcriptional levels of tomato fruit ripening-related genes (such as *PE1*, *PG2*, *LOXB*, and *ACO1*). The results revealed that all ripening-related genes were upregulated in the CR-08 line based on RNA-seq (Fig. 11a–d). To validate the results of RNA-seq, we performed qRT–PCR analysis. The results showed that *PE1* and *PG2* genes, related to fruit wall metabolism, were respectively upregulated by ~5- and 6-fold in the fruits of mutant line CR-08. In addition, the lycopene synthesis-related gene *LOXB* was significantly upregulated in B-stage fruits of the mutant line. The *ACO1* gene, related to ethylene synthesis, was also upregulated in fruits of the mutant line during B stage (Fig. 11e–h).

### Effects of *SlCMT4* knockout on seed and fruit set rate

**Figure 12:**
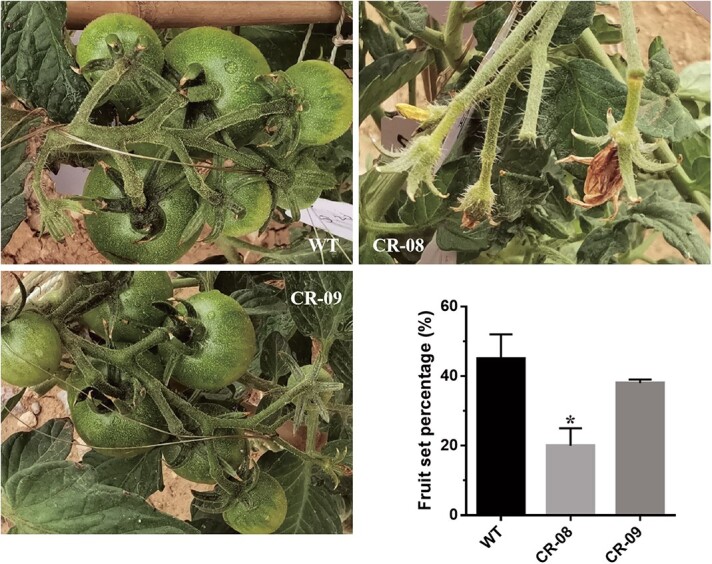
Fruit set rate of WT and mutant lines. Each value represents the mean ± standard deviation of three replicates. The asterisk indicates a significant difference between WT and the CR-08 line. ^*^P < 0.05.

**Figure 13:**
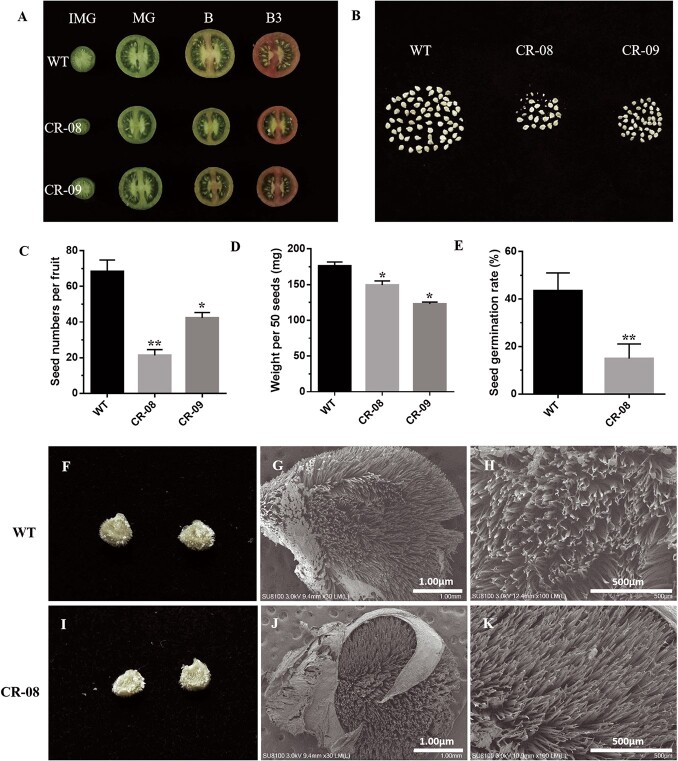
Characteristics of seeds in WT and mutant lines. **a** Transverse sections of fruits at different stages from WT and mutant lines. **b** Digital photographs of seeds of single fruits. **c** Seed number per fruit. **d** Seed weight per 50 seeds at B3 stage. **e** Seed germination rate. **f** and **i** Close-up photographs of seeds; **g**, **h**, **j**, and **k** are scanning electron microscope images (B stage). Data represent the mean ± standard deviation of three replicates. Asterisks indicate statistically significant differences relative to WT, determined using *t*-tests. ^*^P < .05, ^**^P < .01.

The *SlCMT4* mutant lines exhibit a low fruit set rate (Fig. 12). The seed number per fruit (Fig. 13c), weight per 50 seeds (Fig. 13d), and germination rate (Fig. 13e) in the mutant lines decreased; in particular, the seed number per fruit of CR-08 line decreased by ~70%, and some seeds failed to develop normally (Fig. 13a and b). Additionally, the middle of the seeds was sunken (Fig. 13i), and the number of epidermal hair of seeds from the CR-08 line was increased, as confirmed by scanning electron microscopy (Fig. 13j and k).

### *SlCMT4* dysfunction reduces genome-wide cytosine methylation

**Figure 14:**
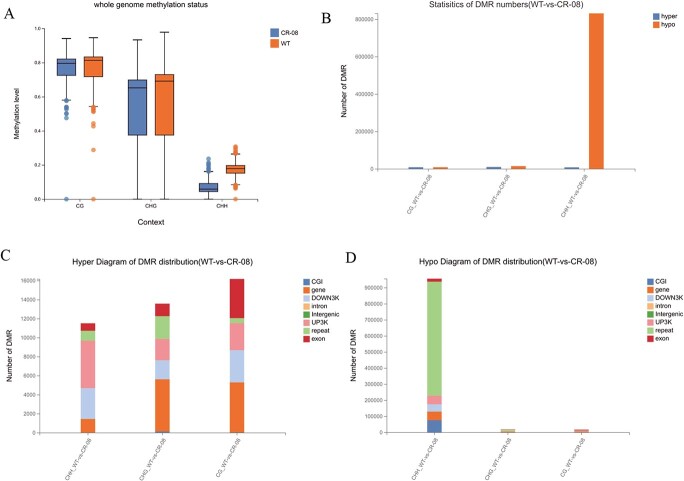
Whole-genome methylation analysis. **a** Cytosine methylation levels of CG, CHG, and CHH in WT and CR-08 mutants. **b**–**d** Statistics of DMR number, hyper diagram of DMR distribution, and hypo diagram of DMR distribution. The *x*-axis represents the difference comparison scheme for each group, and the *y*-axis represents the corresponding difference in DMR number. Different colors represent various component types.

The single-base resolution methylome of *SlCMT4* mutant fruit was investigated by whole-genome bisulfite sequencing (WGBS). The genome-wide CHH methylation level was markedly reduced to 5.82% in *SlCMT4* mutant lines compared with WT plants (Fig. 14a). CHH hypomethylation was observed across each of the 12 chromosomes in *SlCMT4* mutants (Supplementary Fig. S10). The strong reduction in global CHH methylation level is consistent with the role of CMT4 being responsible for CHG and CHH methylation in plants [2]. In addition to genome-wide CHH hypomethylation, *SlCMT4* mutants also exhibited substantially decreased DNA methylation levels in the CHG context (Fig. 14a, Supplementary Fig. S10). On the whole, *SlCMT4* dysfunction displayed a stronger impact on genome-wide DNA methylation compared with WT; gene regions and transposable element (TE) regions both showed CHH hypomethylation on a whole-genome scale in the *SlCMT4* mutant (Supplementary Fig. S11). Thus, disruption of *SlCMT4* function extensively changed the tomato DNA methylome due to integrated regulation of DNA methylation in different cytosine contexts.

Since *SlCMT4* loss of function differentially influences DNA methylation in CG, CHG, and CHH cytosine contexts, we investigated differentially methylated regions (DMRs) of CG, CHG, and CHH separately throughout the genome using DMRcaller [22]. In the CHH context, *SlCMT4* mutants show many more hypo DMRs than hyper DMRs (Fig. 14b). These results are consistent with the assessment of global DNA methylation levels shown in Fig. 14a and Supplementary Figs S10 and S11. That CHH DMRs in *SlCMT4* mutants are overwhelmingly dominated by hypomethylation supports the role of *SlCMT4* as a CHH methylase. We further categorized DMRs into eight types, i.e. CGI, gene, DOWN3K, intron, intergenic, UP3K, repeat, and exon regions. Among the *SlCMT4* mutant CHH hyper DMRs, UP3K, and DOWN3K region accounted for 43.46 and 28.13%, respectively (Fig. 14c). Meanwhile, in the *SlCMT4* mutant CHH hypo DMRs, repeat and CGI regions accounted for 74.18 and 7.82%, respectively (Fig. 14d).

### CRISPR-Cas9-mediated knockout of *SlCMT4* reduces cytosine methylation in the promoter region of candidate genes

**Figure 15:**
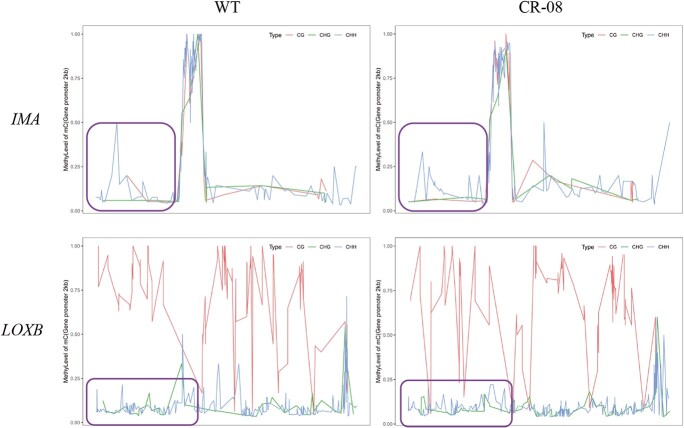
Cytosine methylation levels in the promoter region of candidate genes in WT and CR-08 lines.

We further examined the DNA methylation levels of candidate gene (*IMA* and *LOXB*) target promoters (2 kb) in tomato WT and *SlCMT4* mutant lines. A marked reduction of methylation was observed in a 2-kp region of the *IMA* and *LOXB* promoters in the *SlCMT4*-mutant fruits (Fig. 15).

## Discussion

DNA methylation plays an important function in manipulating many developmental pathways in plants. In this study, we constructed a CRISPR-Cas9 vector of the *SlCMT4* gene, transformed WT tomato, and screened mutants of the *SlCMT4* gene. Based on the phenotypes of mutant lines, some possible functions of the *SlCMT4* gene were characterized at the morphological, physiological, biochemical, anatomical, and molecular levels. The *SlCMT4* mutants showed severe developmental defects with a deletion mutation, including small and thick leaves, increased lateral buds, defective floral organs, small fruit size with reduced setting rate, and defective seed development, suggesting that hypomethylation of plant DNA can cause abnormal development and that DNA methylation may regulate many developmental processes in plants. Similar experiments were performed in *Arabidopsis*, in which antisense-*MET1* lines with decreased methylation exhibited a series of phenotypic and developmental alterations, including smaller plant size, reduced apical dominance, altered leaf size and shape, altered flowering time, and decreased fertility [5, 6]. In addition, *Arabidopsis ddm* mutants with decreased DNA methylation [23] developed morphological abnormalities after several generations of self-fertilization [24].

CRISPR-Cas9-mediated knockout of *SlCMT4* altered tomato plant morphogenesis and promoted lateral branch growth, which was confirmed through the increased number of lateral buds and faster growth rate. Anatomical analysis demonstrated that cortical cells and parenchyma cells in the pith of the lateral buds became larger in transverse and longitudinal sections, and the thickness of the cortical cell layers increased in *SlCMT4* gene mutant lines. In our study, the contents of IAA and tZR in the lateral buds of *SlCMT4* mutant lines increased, while the content of strigol decreased. The above results at hormonal levels were consistent with the multibranching phenotype in the *SlCMT4* mutant plants. In addition, this study found that the mutant lines showed compressed architecture, short internodes, and small and thick leaves, which was confirmed through leaf morphological measurements and anatomical analysis. These results indicate that *SlCMT4* acts as a DNA methyltransferase and plays a critical function in tomato plant morphogenesis.

CRISPR-Cas9-mediated knockout of *SlCMT4* seriously affected the structure and function of tomato flower organs, including defective and everted stamens, and short and thick pistils. Scanning electron microscopy revealed that the stamen cells and pollen grains of the mutant lines were irregular and defective. Pectin methylesterase (PME) is an important enzyme for the modification of side chains of pectin, and is regulated by the pectin methylesterase inhibitor *SlPMEI* gene in tomato [25]. The modification processes of pectin as the main component of the intine will directly influence the development of the intine. The development of the pollen wall is significantly crucial for maintenance of pollen morphology and function. The gene responsible for rapid alkalinization, *SlPRALF*, encodes a preproprotein that negatively regulates pollen tube elongation in tomato [26]. In this study, we further assessed the transcriptional levels of two pollen-specific expression genes (*SlPMEI* and *SlPRALF*) in tomato. The results showed that *PMEI* and *PRALF* genes were respectively upregulated by ~4- and 2.5-fold in pollen of mutant line CR-08. Our results indicate that CRISPR-Cas9-mediated knockout of *SlCMT4* induces the expression of these two pollen-specific genes, which are responsible for irregular and defective pollen. A possible reason is that the upregulated expression of *PMEI* and *PRALF* genes suppressed the development of the pollen wall and pollen tube elongation, resulting in the developmental abnormalities with reduced pollen fertility seen in the CR-08 line.

CRISPR-Cas9-mediated knockout of *SlCMT4* influenced fruit growth and ripening. In our study, the fruit size and weight of the *SlCMT4* mutant lines were reduced compared with WT, which was confirmed through measuring the horizontal diameter, vertical diameter, and weight of fruits at different maturity stages. Besides, transverse anatomical analysis of fruits at the IMG stage displayed reduced seeds in the CR-08 line. Based on the fruit phenotypic alterations mentioned above, we performed a relative transcriptional analysis of the fruit size-related gene *IMA*, which has been reported to play a critical role in tomato fruit growth [27]. The *INHIBITOR OF MERISTEM ACTIVITY* (*IMA*) gene from tomato is involved in multiple regulatory pathways related to cell division, differentiation, and hormone regulation of tomato fruits. In plants overexpressing *IMA*, the number of carpel cells was reduced, the carpels became smaller, and smaller flowers and fruits were produced, indicating that the expression of *IMA* inhibited cell division [27]. Our results showed that the fruit size-related gene *IMA* was upregulated in the fruits of the mutant lines at the IMG stage. Therefore, we deduced that the small-sized fruit phenotype in the mutant lines can be partially explained by reduced seeds and upregulated expression of the *IMA* gene in the mutant lines.

The function of methylation in determining the onset of ripening was first revealed by Zhong *et al*. [28]. They found that treatment of immature tomato fruits with the methyltransferase inhibitor 5-azacytidine induced premature ripening and demonstrated that DNA methylation promotes the regulation of fruit ripening. The binding sites of tomato transcription factor MADS-RIN, related to fruit ripening, are usually located in the demethylation regions of the promoters of many ripening-related genes. The binding of MADS-RIN to these promoters coincides with the demethylation of these sites [28], indicating that DNA methylation is also an important way to regulate fruit ripening. Furthermore, the mature mutant *Cnr* shows a spontaneous epigenetic change caused by methylation of the promoter of the *SBP-CNR* gene [29, 30]. Considering that fruit ripening is related to cell wall alterations [31] and synthesis of carotenoid and ethylene, we further studied the relative transcriptional levels of four ripening-related genes, *PE1* (pectinesterase), *PG2* (polygalacturonase) [33], *LOXB* [34, 35], and *ACO1* [36, 37], in the WT and mutant tomato fruits at B stage. In this study, the *PE1* and *PG2* genes related to fruit wall metabolism were significantly upregulated during the B stage of fruit development in mutant lines. In addition, the lycopene synthesis-related gene *LOXB* was also significantly upregulated at the B stage of fruits of the mutant line. The *ACO1* gene, related to ethylene synthesis, was upregulated during the B stage of fruit development in mutant lines. These results suggest that CRISPR-Cas9-mediated knockout of the DNA methyltransferase gene *SlCMT4* may promote fruit ripening in tomato, which was partially demonstrated by the finding that ripening time of *SlCMT4* RNAi fruits was accelerated by 3–5 days compared with WT fruits in our study (Supplementary Table S1).

In this study, the number and weight of seeds in the mutant lines decreased; in particular, the number of seeds per fruit in the CR-08 line decreased by up to 70% and some seeds failed to develop and mature normally. The middle of the seeds in the CR-08 line was sunken, and the epidermal hairs of the seeds in CR-08 line were seen by scanning electron microscopy to be increased. We performed pollen observation by scanning electron microscopy of the WT and mutant lines. The results revealed that most pollen grains in the CR-08 line were aberrant and defective, suggesting that knockout of *SlCMT4* significantly affected pollen grain development and led to the formation of defective pollen grains. Additionally, *SlCMT4*-knockout stamens and pistils were twisted and shorter, respectively. These changes in stamens, pollen grains, and pistils reduced the success of pollination. Therefore, the mutant line CR-08 had a lower fruit set rate. In angiosperms, branching patterns greatly determine plant architecture and affect nutrient allocation, height, light-harvesting efficiency, and visibility for pollinators [38]. Thus, vigorous vegetative growth of the *SlCMT4* mutants probably accounts for the low fruit set percentage.

DNA methylation regulates gene expression in different parts of plants and at different developmental stages, leading to some variations in plant morphologies. In this study, a reduction of methylation was observed in a 2-kp region of the *IMA* and *LOXB* promoters in the *SlCMT4*-mutant fruits, indicating that the hypermethylation status of the CHH context is critical for inhibition of *IMA* and *LOXB* promoter activity. The reduction in methylation of these residues causes an increase in *IMA* and *LOXB* expression. This study clarified that *SlCMT4* can cooperate in the epigenetic regulation of tomato fruit growth, development, and ripening by regulating the methylation patterns of key transcription factors.

## Materials and methods

### CRISPR-Cas9 knockout of *SlCMT4*

CRISPR-Cas9 was used to select two specific sgRNAs that targeted tomato *SlCMT4* (Fig. 1). The oligo dimer including the gRNA-U6 fragment amplified was linked to the CRISPR-Cas-BGK012-DSG vector (15 250 bp). The resulting BGK012-DSG-*SlCMT4* vector (Supplementary Fig. S12) was transformed into WT tomato AC^++^ using stable *Agrobacterium tumefaciens*. Genomic DNA was extracted from young leaves of transformed plants and amplified by PCR using primers flanking the target sites. The PCR products were sequenced to identify mutations. The primer sequences were as follows: F, 5′-AATTAGCTCTGTTTTACCCTCAA-3′; R, 5′-CTGCTTCCTCACACTTTTCTCTG-3′.

### Measurement of organ architecture parameters

To characterize the differences in organ morphology between WT and mutant lines, we counted and measured the number, length, and diameter of lateral branches as well as the length, width, perimeter, and area of compound leaves and internode length from WT and mutant lines. The length, width, perimeter, and area of compound leaves from 3-month old plants were determined with a leaf area meter (Yaxin-1241, Beijing). The length and diameter of lateral branches were measured after 5 days of growth. Fruit size and weight were measured using a Vernier caliper (0–150 mm, Shanghai) and electronic balance (0–100 g, Shanghai). The fruit set of WT and mutant lines was also determined.

### Anatomical and cytological analyses of lateral branches and leaves

The 5-day-old lateral branches and mature leaves collected from WT and mutant lines were immediately fixed with 2.5% glutaraldehyde. The experimental steps for tissue sectioning and staining were as follows: (i) dewaxing and rehydration; (ii) Safranin O staining; (iii) decolorization; (iv) fixed green staining; (v) placing sections into three cylinders of xylene for 5 minutes; and (vi) finally mounting the tissue sections with neutral balsam. The sections were observed under a microscope (Eclipse E100, Nikon, Japan) fitted with an image acquisition system (DS-U3, Nikon, Japan). The cell morphologies of lateral branches and leaves were measured and visualized using CaseViewer 2.3 software and photographed. The number and size of cells were estimated with ImageJ (an image-analyzing program).

### Scanning electron microscopy

Flower organs (stamens, pistils, and pollen grains) were collected from the inflorescences of WT and mutant lines at anthesis. Samples were fixed in 2.5% glutaraldehyde for 2 hours and subsequently dehydrated in an ethanol–water series. After dissection, the samples were dried in a critical point dryer and sputtered with gold for 30 seconds. The samples were observed and images were taken using a scanning electron microscope (SU8100, Hitachi, Japan).

### Quantitative real-time PCR analysis

Total RNA was extracted from WT and mutant lines using an RNeasy Plant Mini Kit (Qiagen,74904), and RNA concentration was detected using a NanoQuant infinite M200PRO (TECAN). Reverse transcription was performed using using AMV (200 U/μl) reverse transcriptase (Invitrogen). qRT–PCR was carried out according to a previous method [39]. The tomato *SlCAC* gene was used as an internal standard [40]. Relative gene expression levels were quantified by the 2$ -\bigtriangleup\bigtriangleup C $*T* method [41]. The primers *SlCMT4*-F and *SlCMT4*-R were used to determine the expression levels of *SlCMT4* in WT and mutant lines. All qPCR primers designed by Primer Express Software v2.0 are shown in Supplementary Table S2.

### Extraction and quantification of plant hormones

Lateral branches were collected from 3-month-old plants (1.0 g per plant) and homogenized in liquid nitrogen. Extraction and quantification of plant hormones followed the methods reported by Kojima *et al*. [42] and Pan *et al*. [43].

### Whole-genome bisulfite sequencing and data analysis

Genomic DNA was extracted from the pericarp tissues of WT and CRISPR line #08 fruits, and bisulfite treatment was implemented using the Zymo EZ DNA Methylation-Gold Kit. The raw sequencing data were filtered using SOAPnuke software [44]. Clean data were aligned to the tomato reference genome (NCBI version GCF_000188115.4_SL3.0) using Bismark software [45], and statistical information such as the comparison rate and bisulfite conversion rate of each sample was calculated. Bismark with default parameters was also used to remove duplicated sequence reads. Differentially methylated regions (DMRs) refer to certain DNA fragments in different samples that exhibit different methylation patterns in the genome. The DNA methylation level is the ratio of the number of reads that support methylation to the number of reads covering the site [46] and was calculated as follows: *Rm_average_* = *Nm_all_*/(*Nm_all_* + *Nnm_all_*) × 100%. DMRcaller [22] R was used to calculate and analyze DMRs. We used BEDTools [47] to calculate DMR-associated genes or other genome elements based on position. By default, a 1-bp overlap between DMR and genes or other elements is the DMR-associated gene or DMR-associated element. Furthermore, we performed GO enrichment and Kyoto Encyclopedia of Genes and Genomes (KEGG) pathway enrichment analysis on these related genes.

## Supplementary Material

Web_Material_uhac081

## Data Availability

Raw transcriptome sequencing data reported in this paper have been deposited (PRJCA007796) in the Genome Sequence Archive in the BIG Data Center, Chinese Academy of Sciences, under accession code CRA005782 at https://ngdc.cncb.ac.cn/.
