# Supplementary material for: CRISPR/Cas9-targeted mutagenesis of SlCMT4 causes changes in plant architecture and reproductive organs in tomato
Source: Hortic Res. 2022 Apr 11;9:uhac081. doi: 10.1093/hr/uhac081 (PMC9233168; doi:10.1093/hr/uhac081)
Supplement: Web_Material_uhac081 [file web_material_uhac081.pdf]

Title: CRISPR/Cas9-targeted mutagenesis of *SlCMT4* causes changes in plant architecture and reproductive organs in tomato

Author: Xuhu Guo[1,2*], Jianguo Zhao[3], Zhiwen Chen[3], Jun Qiao[3], Yongfang Zhang[1], Hong Shen[1], Zhongli Hu[4*]

1. School of Life Sciences, Shanxi Datong University, Datong 037009, China.

2. Protected Agricultural Technology Research and Development Center, Shanxi Datong University, Datong 037009, China.

3. Institute of Carbon Materials Science, Shanxi Datong University, Datong, 037009, China

4. Laboratory of Molecular Biology of Tomato, Bioengineering College, Chongqing University, Chongqing 400044, China.

Corresponding author. E-mail: xhguo201010@126.com (Xuhu Guo); huzongli@cqu.edu.cn (Zongli Hu)

Material and methods

Plant materials and growth conditions

In this study, a near-isogenic tomato line (*Solanum lycopersicum* 'Ailsa Craig' AC^{++}) was used as WT. Wild-type and mutant plants grown under greenhouse conditions (16-h-day/8-h-night cycle, 25ºC/18°C day/night temperature, 80% humidity, and 250 μmol m^{-2} s^{-1} light intensity). Plants of the first generation (T0) came from cotyledon callus, and plants of the second generation (T1) from seedlings, which were used in our experiments. Flowers were labeled at anthesis to count the ripening time of tomato fruits. The ripening of tomato fruits were divided into different stages according to the days post anthesis (DPA) and fruit color. In WT, fruits of 20 DPA were identified as immature green (IMG) fruits. Fruits of 35 DPA were defined as mature green (MG) fruits with no obvious color change. Fruits of 38 DPA were defined as breaker (B) fruits with the color change from green to yellow. In addition, other stages fruits of 3 d after breaker and 7 d after breaker were also used. The plant samples collected were immediately frozen with liquid nitrogen and stored at −80ºC.

Construction of *SlCMT4* RNAi vector and plant transformation

A 442-bp specific DNA fragment of *SlCMT4* was amplified from WT flower cDNA with the following primers: Forward, 5′-GGTACCAAGCTTGAGGTCGCCGAGATTGGT-3′ and Reverse, 5′-CTCGAGTCTAGATGAATTGCTCTCAGCCGTTA-3′, which had been tailed with KpnI/HindIII and XhoI/XbaI restriction sites at the 5′ end, respectively. Then, the amplified products were digested with HindIII/XbaI and KpnI/XhoI and inserted into the pHANNIBAL plasmid at the HindIII/XbaI restriction site in the sense orientation and at the KpnI/XhoI restriction site in the antisense orientation. Finally, the double-stranded RNA expression unit, containing the cauliflower mosaic virus (CaMV) 35S promoter, *SlCMT4* antisense fragment, PDK intron, *SlCMT4* sense orientation fragment, and OCS terminator, was linked to the plant binary vector pBIN19 with SacI and XbaI restriction sites. The generated binary plasmids were transformed into WT tomato cotyledon explants via *Agrobacterium* strain LBA440469 [1]. Positive transgenic lines were screened for kanamycin (50mg L^{-1}) resistance. The transgenic lines were detected with primers NPTII-F (5′-GACAATCGGCTGCTCTGA-3′) and NPTII-R (5′-AACTCCAGCATGAGATCC-3′). Total RNA was extracted from flowers of WT and transgenic plants to confirm the silencing efficiency of *SlCMT4* in the transgenic lines. Quantitative

real-time PCR (qRT-PCR) results showed that *SlCMT4* transcripts were markedly reduced in 10 independent transgenic plants compared with WT. Among which, the accumulation of *SlCMT4* transcripts in lines 4, 10 and 11 was remarkably reduced to roughly 20–30% of control levels (Fig. S13). Thus lines 4, 10 and 11 with the strongest down regulation were selected for further study.

RNA-Seq and data analysis

According to the manufacturer's instructions, total RNA was extracted from the pericarp tissues of WT and CRISPR line #08 fruits at different stages (IMA, MG and B) using the RNAprep pure plant kit (TIANGEN, Shanghai, China). A total of 1 μg purified mRNA was used for cDNA library construction using the NEBNext UltraTM RNA Library Prep Kit for Illumina (NEB, USA). Illumina sequencing was performed using the Illumina NovaSeq platform. The raw data were mapped to the tomato reference genome SL4.0 using HISAT2 [2], and differentially expressed genes (DEGs) analysis was performed using DEseq2 based on the negative binomial distribution [3]. Gene expression levels were determined as fragments per kilobase of transcript per million fragments mapped (FPKM), using the following formula: FPKM = {cDNA Fragments\over {Mapped Fragments (Millions) *Transcript Length(kb)}}. Genes with an adjusted p-value < 0.05 and 1.5-fold or greater expression change identified by DESeq2 were considered differentially expressed. Three biological replicates were performed for both the WT and CR-08 lines.

References

1. Chen G, Alexander L, Grierson D. Constitutive expression of EIL-like transcription factor partially restores ripening in the ethylene-insensitive *Nr* tomato mutant. *J Exp Bot*. 2004;**55**:1491-97.
2. Kim D, Langmead B, Salzberg SL. HISAT: a fast spliced aligner with low memory requirements. *Nat methods*. 2015;**12**:357-60.
3. Love MI, Huber W, Anders S. Moderated estimation of fold change and dispersion for RNA-seq data with DESeq2. *Genome Biol*. 2014;**15**:550.

Supplementary Tables

Table S1. Days from anthesis to breaker stage for control and *SlCMT4*-silenced lines

Tomato Line	Days
Wild type	38.0±0.5
RNAi 4	32.3±0.5
RNAi 10	35.5±0.6
RNAi 11	35.5±0.6

Table S2. qPCR primers used in this study

Primers	Primer sequences (5' → 3')
SlCMT4-F	CTTGGCACAAAACTCTCTGGTC
SlCMT4-R	TTCTCAACACCTTCATTCCTAACAT
SlCAC-F	CAGGAAGGTGTCCGGTCATC
SlCAC-R	TAAACAAGACCCTCCCTGCG
PMEI-F	GTCAAAACGTACCGGCATTT
PMEI-R	GGTGCACTTGAAGCCAATCT
PRALF-F	CGATGTCGAAGAACGCCATC
PRALF-R	CGACGTTGGCATCTGGTGAT
IMA-F	CATGCTGCTAGTGTCGGTGG
IMA-R	CCTTCTGTGGAAATTGCGGT
PE1-F	CCTTCCGCTCTGCCACTCTT
PE1-R	CCAACTCGAAGTGCCACTGC
PG2-F	ACTTGTGGTCCAGGTCATGG
PG2-R	GATCCTCCCTGCCAAGTCTT
LOXB-F	CATAAGAGTTGGAAGAACAG
LOXB-R	TACAACACAACACACAAGAC
ACO1-F	TATATGCTGGACTCAAGTTTC
ACO1-R	TAATTTCACTAGAAGGCACC

Supplementary Figures

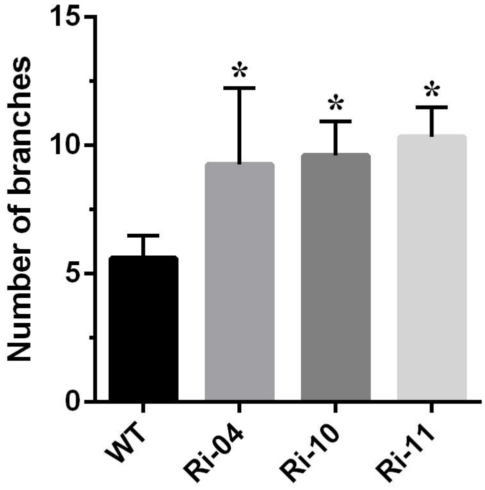

Fig. S1 Lateral branch number in WT and *SlCMT4*-RNAi lines (two-month-old). Data are means of three biological replicates ± SD. Asterisks indicate statistically significant differences relative to the WT and were determined using t-tests. *, $p < 0.05$.

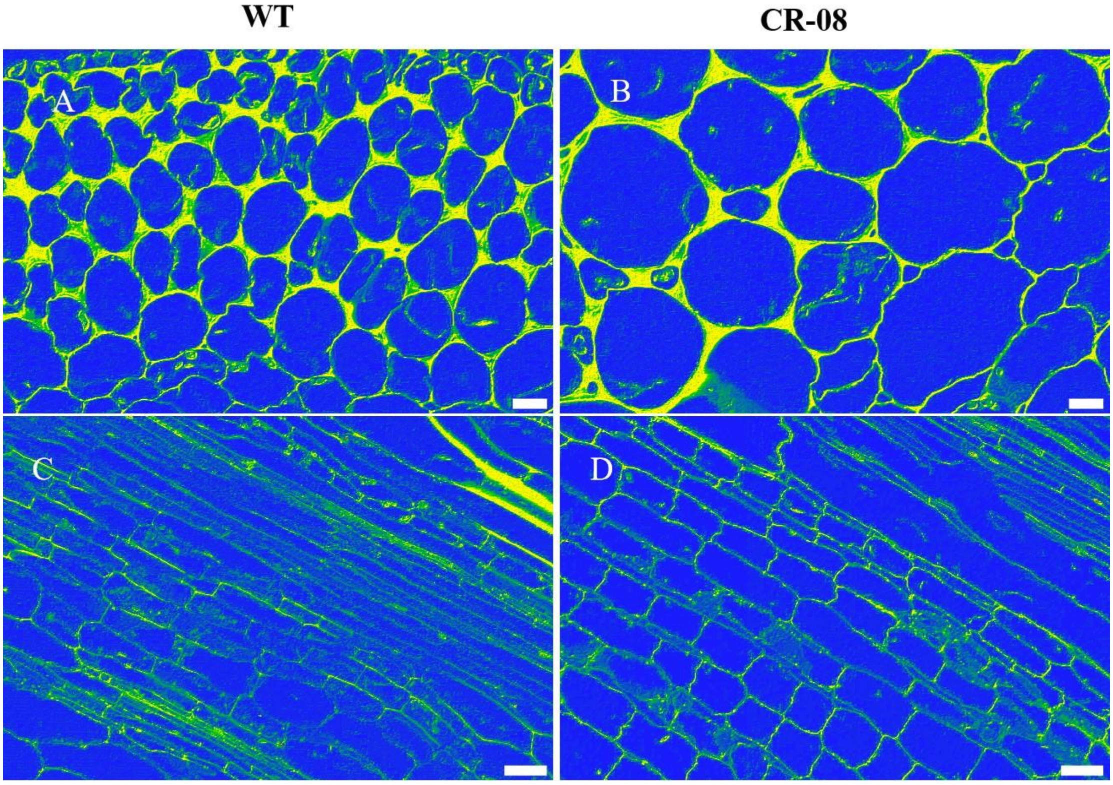

Fig. S2 The anatomical analysis of the lateral buds from WT (A, C) and CR-08 (B, D) plants. (A, B) represent the cortical cells from transverse section of lateral buds. Bars = 20 μm. (C, D) represent the

cortical cells from longitudinal section of lateral buds. Bars = 50 μm. The samples were prepared from 5-day-old lateral buds.

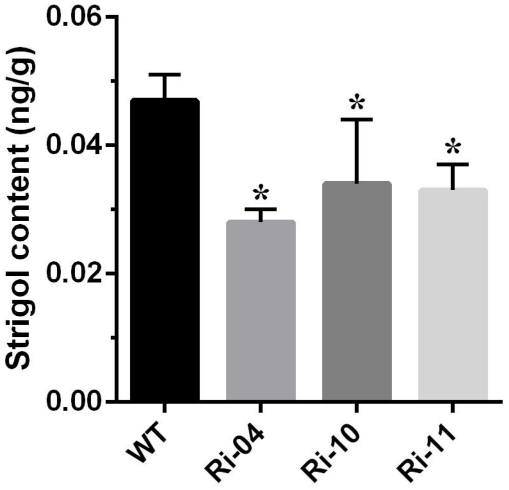

Fig. S3 The content of strigol in lateral buds of WT and *SlCMT4*-RNAi lines. Data are means of three biological replicates ± SD. Asterisks indicate statistically significant differences relative to WT and were determined using t-tests. *, $p < 0.05$.

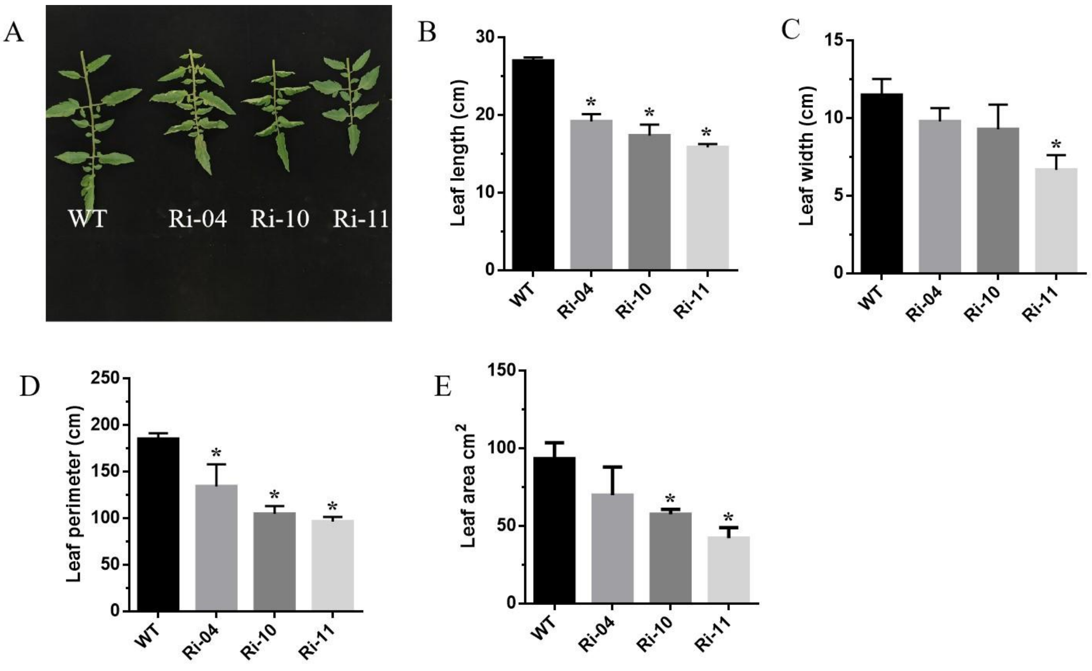

Fig. S4 Leaf morphologies of WT and *SlCMT4*-RNAi lines. (A) Digital photograph of leaves from WT and *SlCMT4*-RNAi lines. (B)-(E) represents the length, width, perimeter and area of leaves from WT and *SlCMT4*-RNAi lines, respectively. Data are means of three biological replicates ± SD. Asterisks

indicate statistically significant differences relative to WT and were determined using t-tests. *, $p < 0.05$.

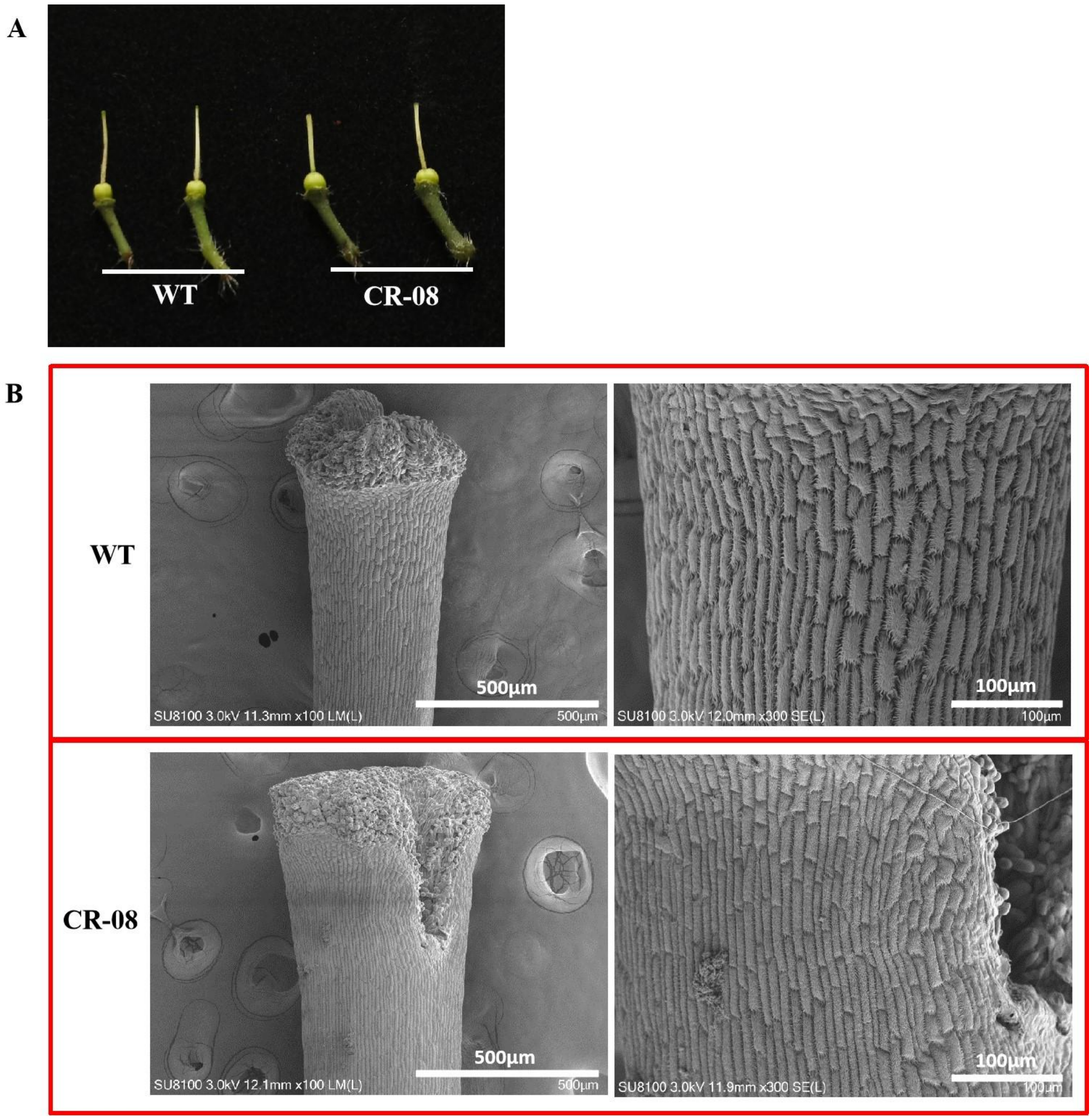

Fig. S5 Phenotypes of the pistils in CR-08 lines. (A) Digital photograph and (B) scanning electron microscopy images.

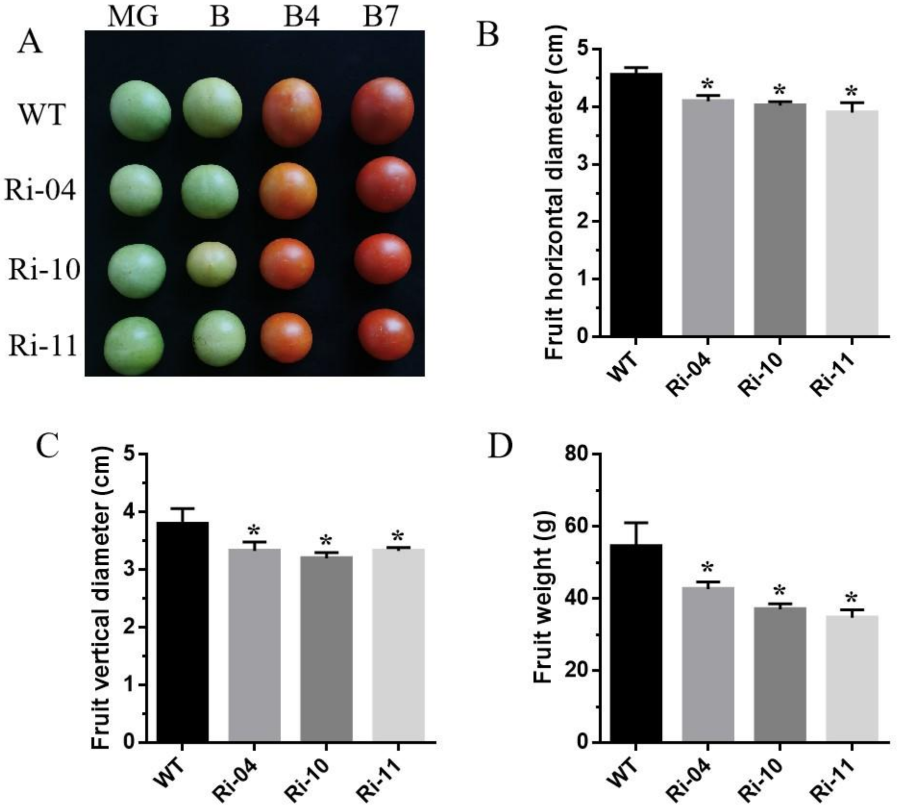

Fig. S6 Phenotypes of fruits from the *SlCMT4*-RNAi lines. (A) Digital photograph of fruits at different stages from WT and transgenic lines. (B-D) represents the horizontal diameter, vertical diameter and weight of fruit at different stages from WT and RNAi lines respectively. MG, B, B4 and B7 represent mature green fruit, breaker fruit, 4 days after breaker fruit and 7 days after breaker fruit respectively.

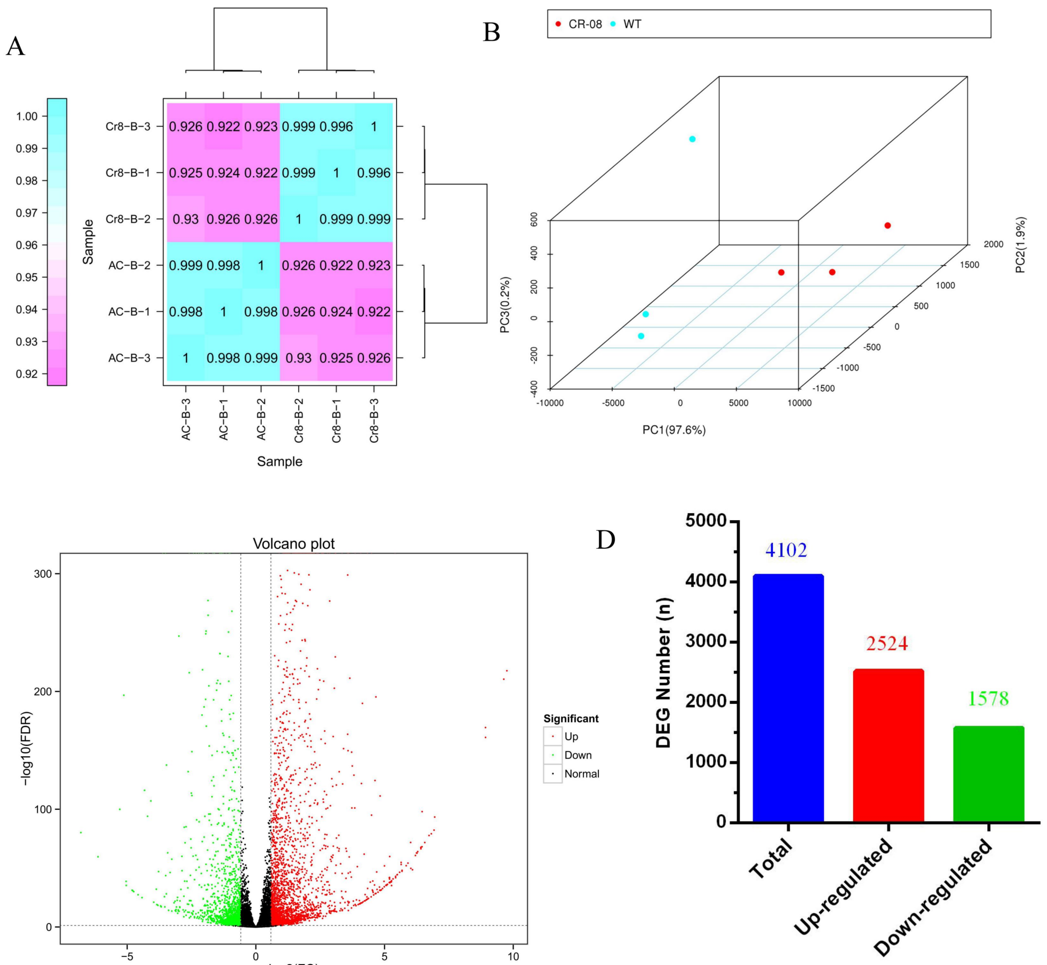

Fig. S7 Overview of tomato pericarp transcriptome in WT and CR-08 lines. (A) Pearson correlation coefficient (PCC) of analysis of all genes between the six samples. (B) Principal component analysis of all samples. Red and light blue colors represent the samples of CR-08 and WT, respectively. (C) Volcano plot of differentially expressed genes. (D) represents the total number of up-regulated and down-regulated genes.

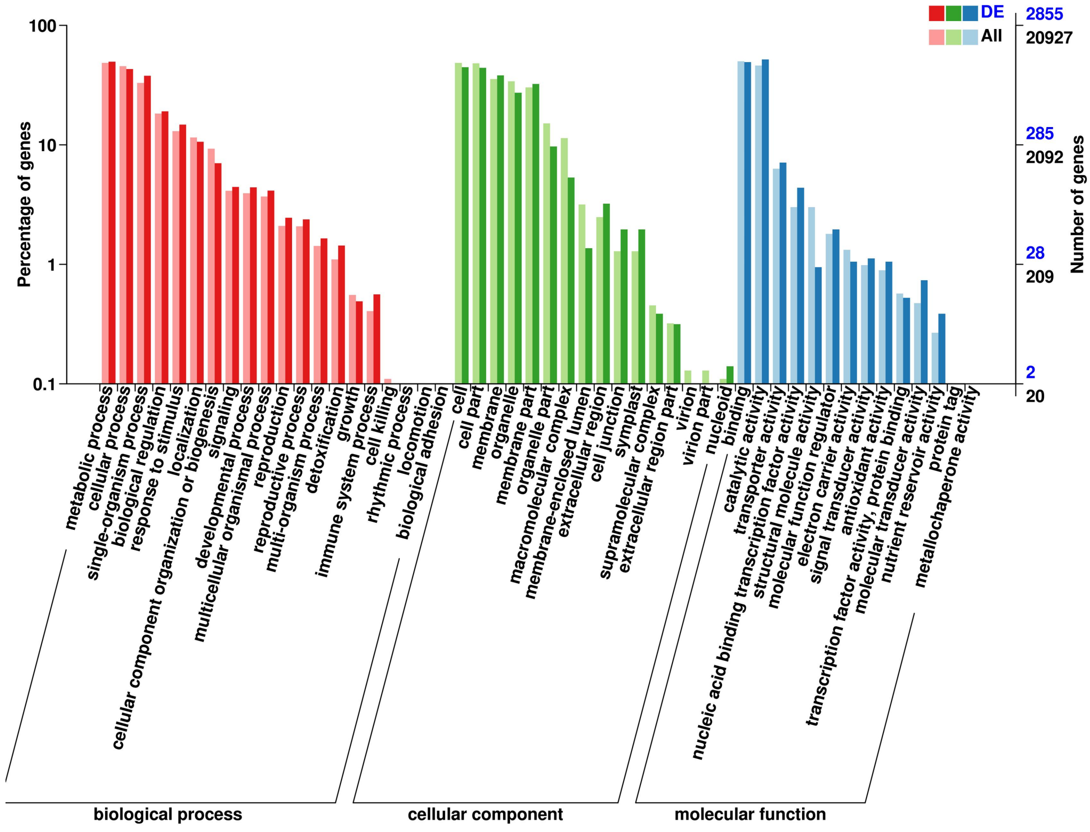

Fig. S8 GO enrichment analysis of DEGs. The X-axis represents three biological functions of these DEGs, namely molecular function, biological process, and cellular component. The Y-axis represents percentage (left) or number (right) of genes categorized into different functional pathways.

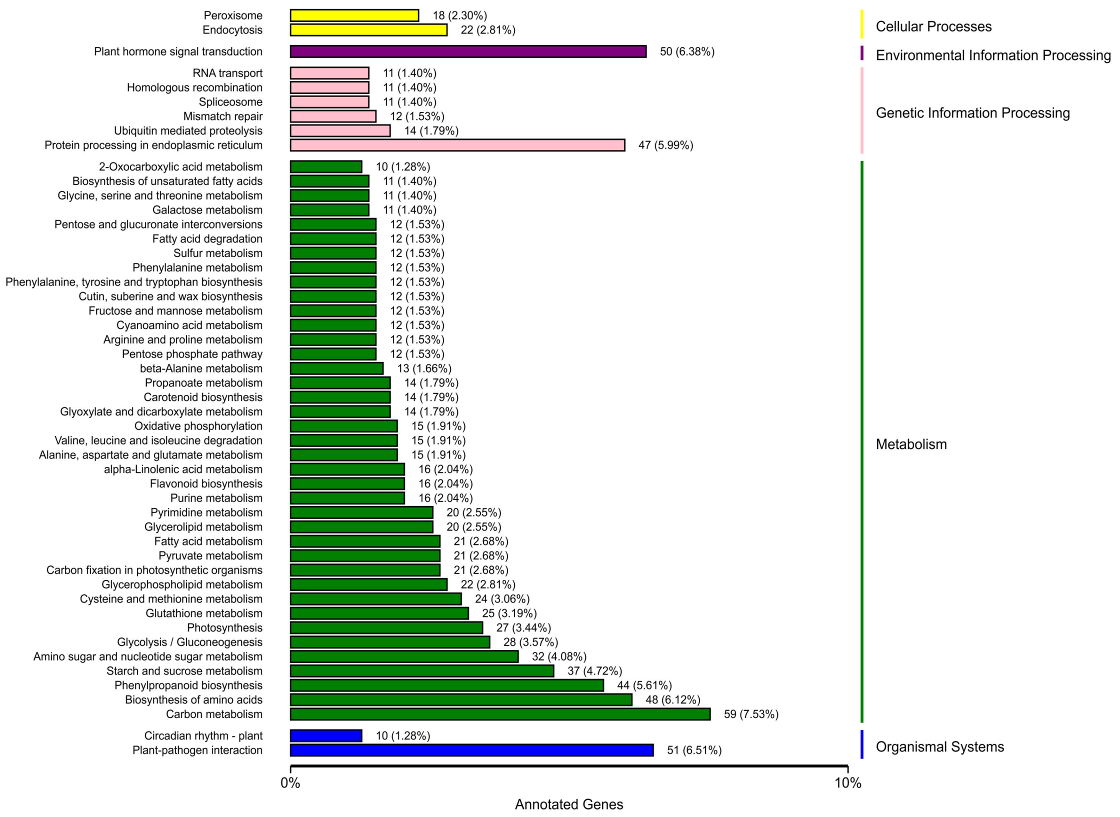

Fig. S9 KEGG pathway analysis for identification of the differentially expressed genes functional categorization.

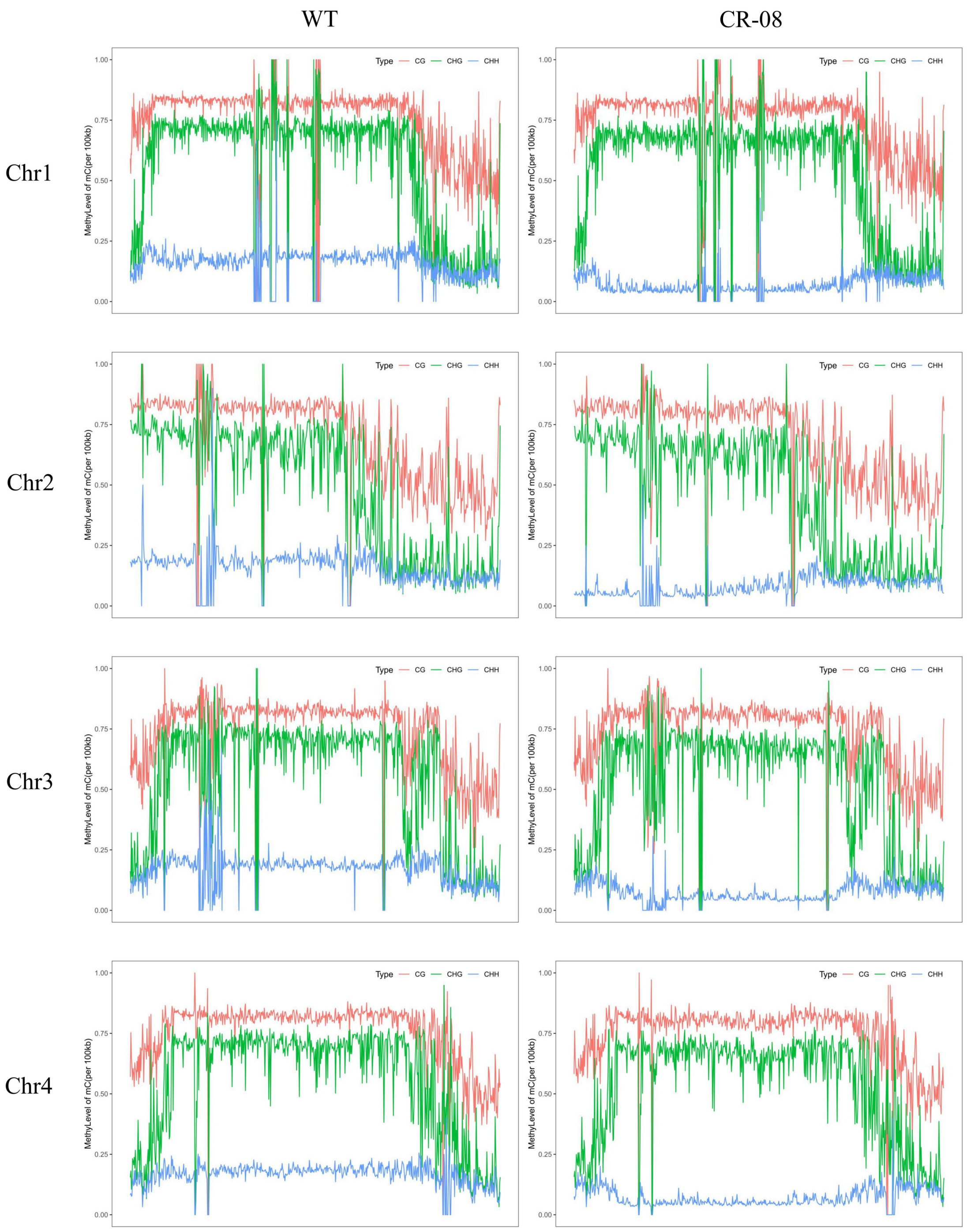

Fig. S10 Cytosine methylation levels were calculated per 100 kb in all the 12 chromosomes in the WT and CR-08 mutants. The horizontal axis indicates chromosomal positions, and the vertical axis represents methylation levels of cytosine in WT and mutant lines. Cytosine levels in the CG, CHG, and CHH contexts are indicated by red, green, and blue colors, respectively.

WT CR-08

Chr5

Chr6

Chr7

Chr8

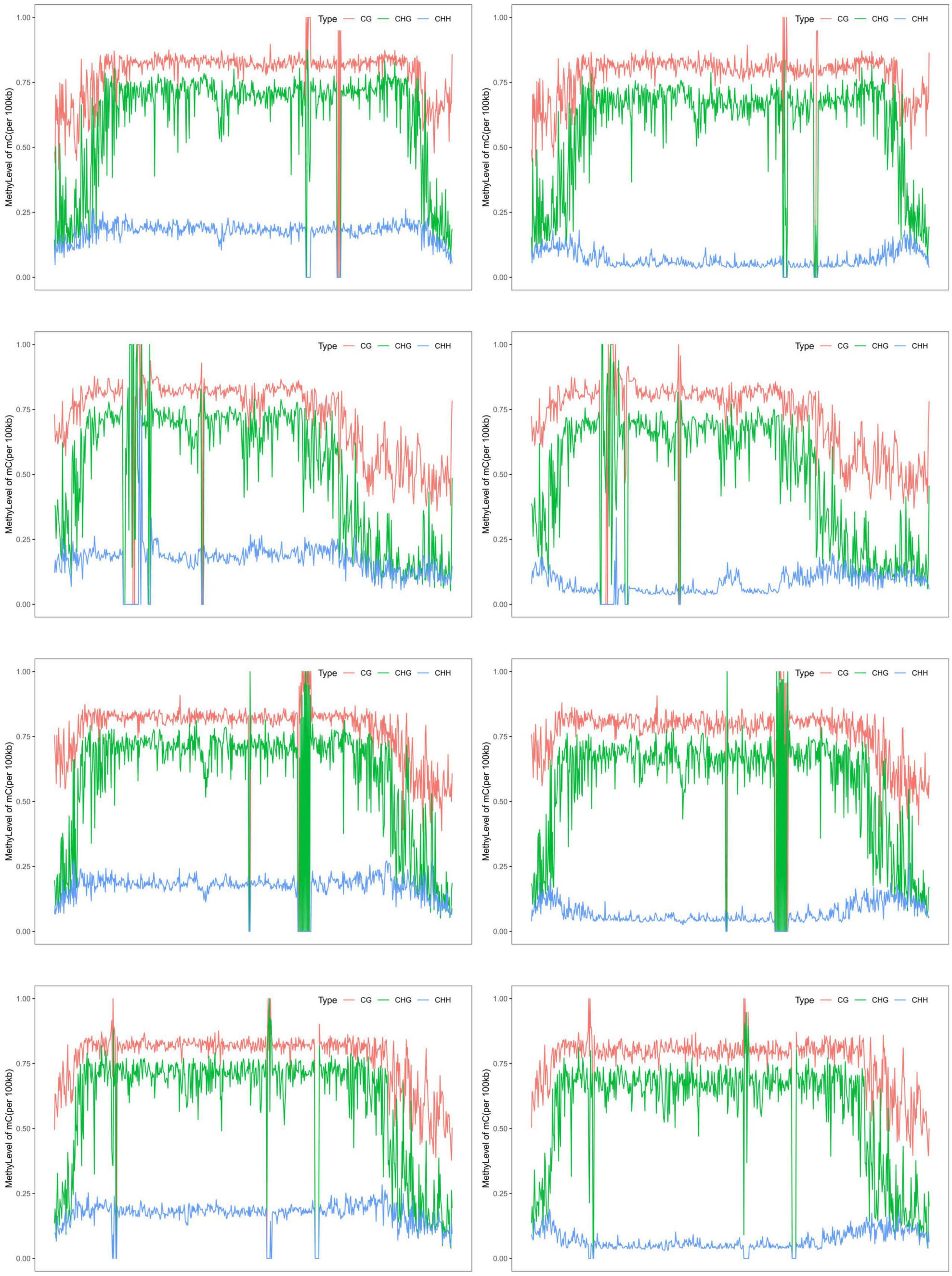

Fig. S10 Continued

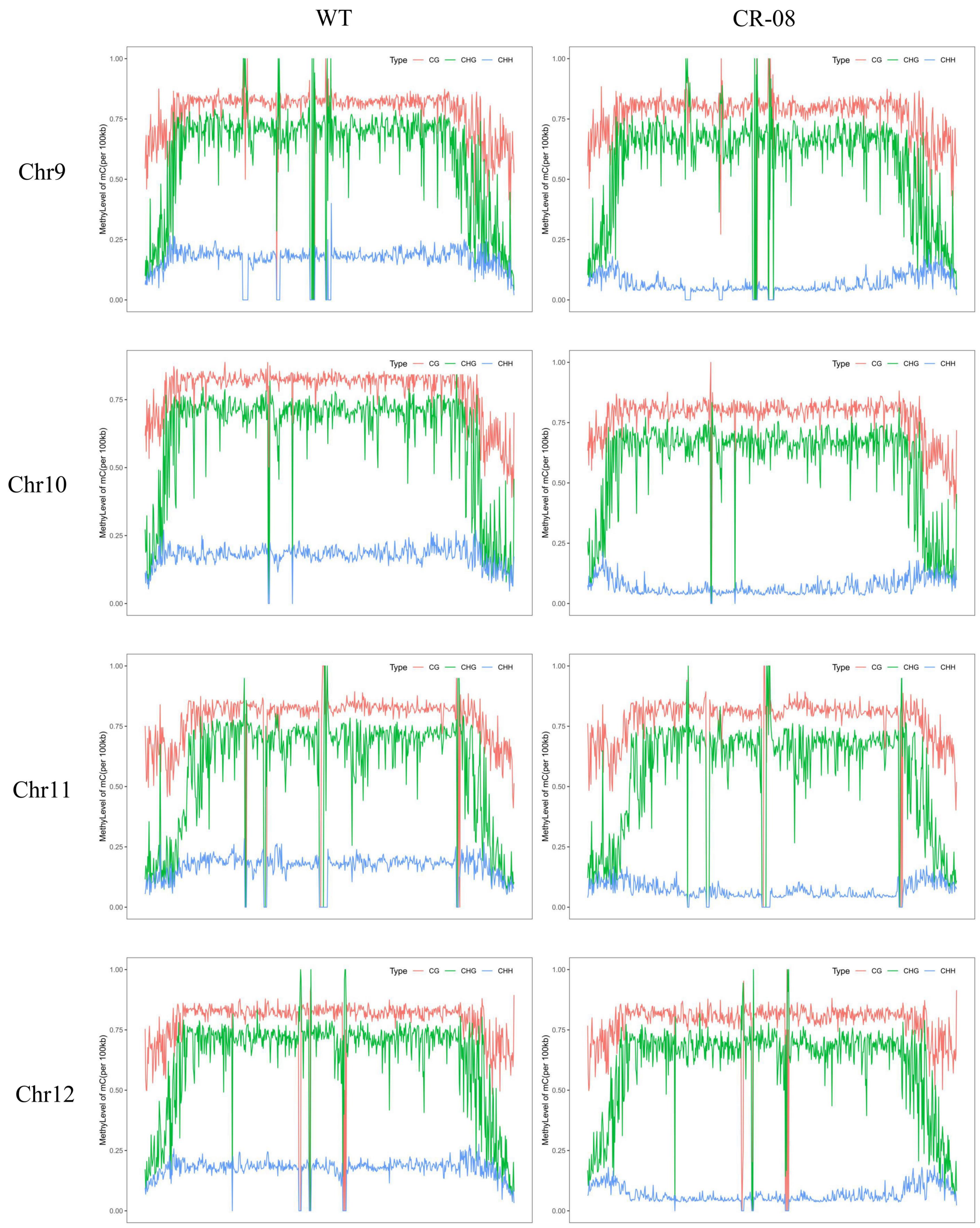

Fig. S10 Continued

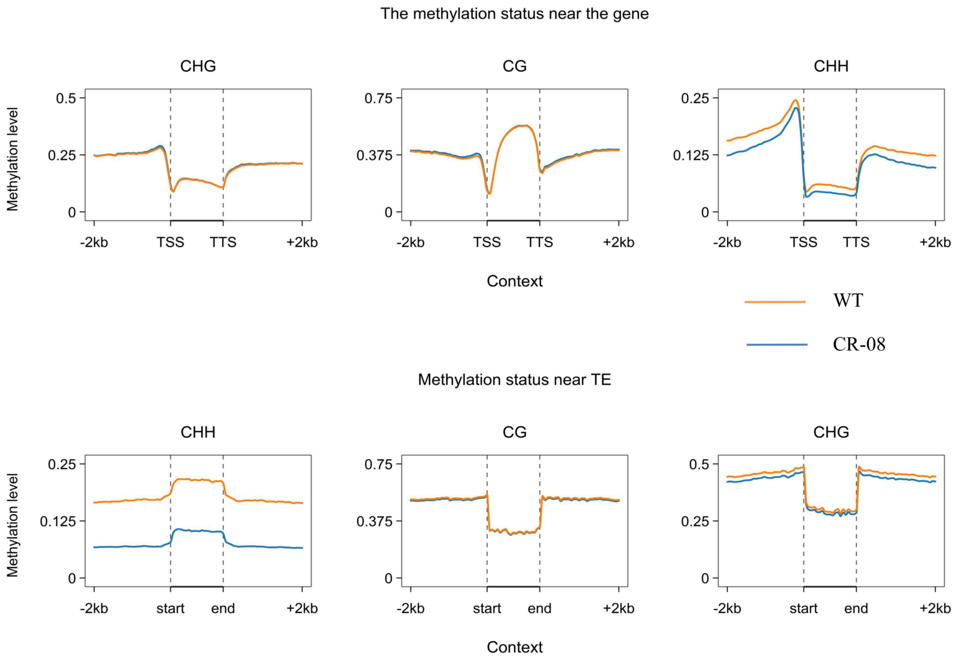

Fig. S11 The methylation status near the gene and TE regions, each with their contiguous 2kb upstream and downstream flank regions. The horizontal axis is the location of the gene region, and vertical axis is the methylation level of sample.

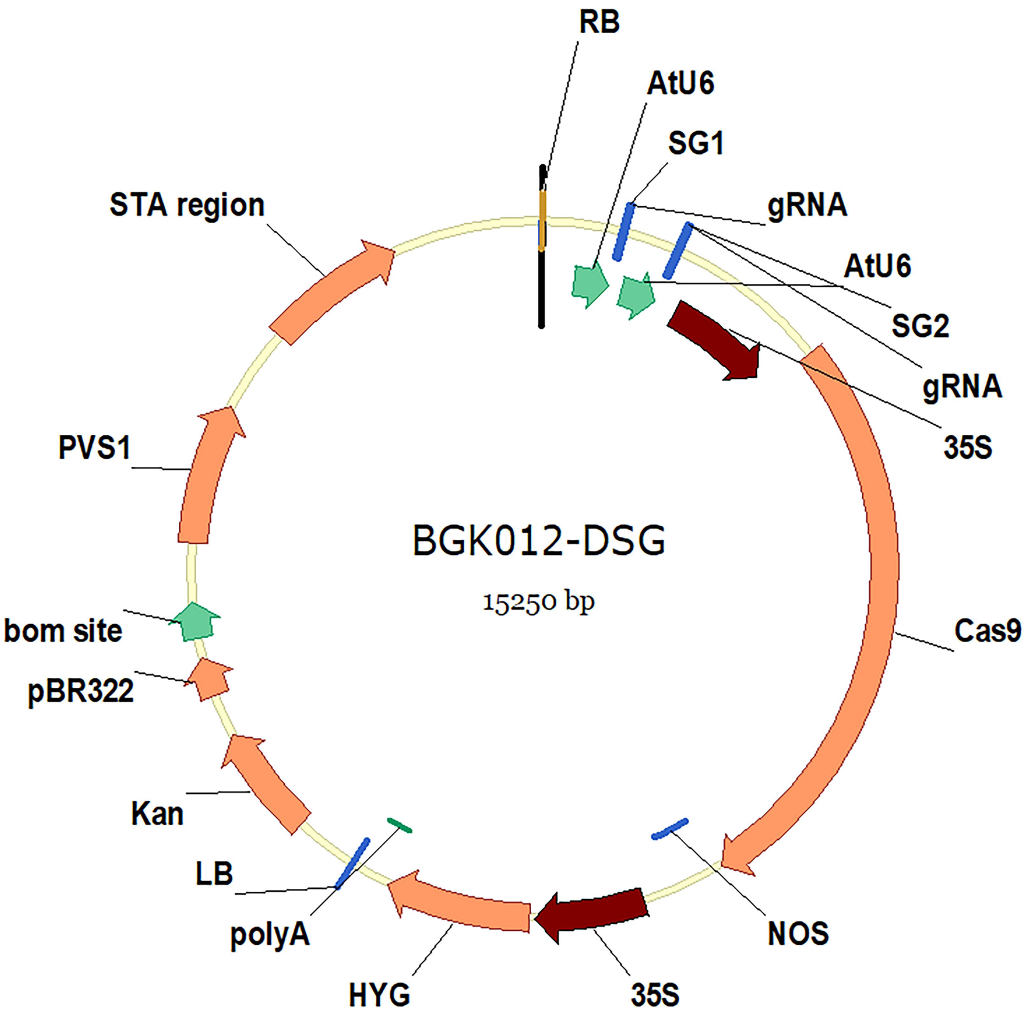

Fig. S12 The resulting BGK012-DSG-SlCMT4 vector

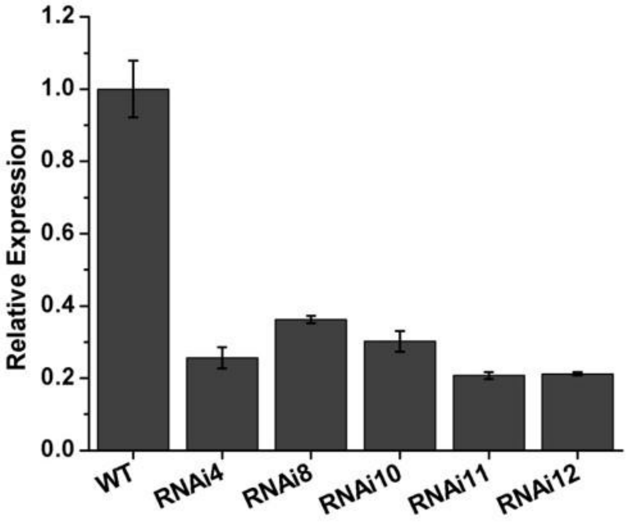

Fig. S13 Expression profiles of *SlCMT4* between WT and RNAi lines. The expression data of WT plants were normalized to 1. Each value represents the mean± SD of three replicates.
